# New Titanocene (IV) Dicarboxylates with Potential Cytotoxicity: Synthesis, Structure, Stability and Electrochemistry

**DOI:** 10.3390/ijms24043340

**Published:** 2023-02-07

**Authors:** Dmitry A. Guk, Karina R. Gibadullina, Roman O. Burlutskiy, Kirill G. Pavlov, Anna A. Moiseeva, Viktor A. Tafeenko, Konstantin A. Lyssenko, Erik R. Gandalipov, Alexander A. Shtil, Elena K. Beloglazkina

**Affiliations:** 1 Chemistry Department, Lomonosov Moscow State University, 1/3 Leninskie Gory, 119991 Moscow, Russia; 2 International Institute of Solution Chemistry of Advanced Materials and Technologies, ITMO University, 9 Lomonosov Street, 191002 Saint-Petersburg, Russia; 3 Blokhin National Medical Research Center of Oncology, 24 Kashirskoye Shosse, 115522 Moscow, Russia; 4 Institute of Cyber Intelligence Systems, National Research Nuclear University MEPhI, 31 Kashirskoye Shosse, 115409 Moscow, Russia

**Keywords:** titanocene, carboxylate ligands, biogenic metals, redox activity, cytotoxicity

## Abstract

The search for new anticancer drugs based on biogenic metals, which have weaker side effects compared to platinum-based drugs, remains an urgent task in medicinal chemistry. Titanocene dichloride, a coordination compound of fully biocompatible titanium, has failed in pre-clinical trials but continues to attract the attention of researchers as a structural framework for the development of new cytotoxic compounds. In this study, a series of titanocene (IV) carboxylate complexes, both new and those known from the literature, was synthesized, and their structures were confirmed by a complex of physicochemical methods and X-ray diffraction analysis (including one previously unknown structure based on perfluorinated benzoic acid). The comprehensive comparison of three approaches for the synthesis of titanocene derivatives known from the literature (the nucleophilic substitution of chloride anions of titanocene dichloride with sodium and silver salts of carboxylic acids as well as the reaction of dimethyltitanocene with carboxylic acids themselves) made it possible to optimize these methods to obtain higher yields of individual target compounds, generalize the advantages and disadvantages of these techniques, and determine the substrate frames of each method. The redox potentials of all obtained titanocene derivatives were determined by cyclic voltammetry. The relationship between the structure of ligands, the reduction potentials of titanocene (IV), and their relative stability in redox processes, as obtained in this work, can be used for the design and synthesis of new effective cytotoxic titanocene complexes. The study of the stability of the carboxylate-containing derivatives of titanocene obtained in the work in aqueous media showed that they were more resistant to hydrolysis than titanocene dichloride. Preliminary tests of the cytotoxicity of the synthesised titanocene dicarboxilates on MCF7 and MCF7-10A cell lines demonstrated an IC50 ≥ 100 μM for all the obtained compounds.

## 1. Introduction

According to WHO statistics, oncological diseases remain one of the main causes of death in developed and developing countries, ahead of other chronic diseases and diseases of the cardiovascular system [1]. Platinum-based drugs originating from the discovery of the antitumor properties of cisplatin in 1976 [2] remain the most common choice in terms of metal-based anticancer therapy; however, a high risk of undesirable side effects due to the accumulation of abiogenic platinum [3,4] (the same applies to other abiogenic metals, for example, iridium) [5] in the patient’s body and the rapid development of tumor resistance to platinum-containing drugs [6] make the search for new cytotoxic coordination compounds of other transition metals an urgent task in modern bioinorganic and medicinal chemistry.

Cytotoxic coordination compounds based on biogenic metals, which have a lower overall toxicity than abiogenic platinum derivatives, have attracted considerable attention [7]. In particular, titanocene dichloride was tested as an agent for the treatment of metastatic breast and colon tumors and pre-clinical trials were successfully completed in 1993–2000 [8,9].

In the course of laboratory and preclinical tests, it was shown that titanocene dichloride has a pronounced cytotoxicity on different cell lines, such as adenocarcinoma of the lung, breast, and gastrointestinal tract [8,10]. It was also shown that titanocene dichloride has a pronounced cytotoxicity on platinum-resistant cell lines, which indicates a different mechanism of action to cisplatin [11]. In vivo studies of titanocene dichloride as a therapeutic agent for the treatment of Ehrlich ascites carcinoma inoculated into xenograft mice resulted in an 80% cure, with no noticeable side effects associated with drug toxicity [8]. In addition, the final product of titanocene dichloride metabolism is titanium dioxide, an inert and fully biocompatible substance widely used in medicine and the food industry, which is an FDA-approved drug excipient.

Additionally, regarding titanocene dichloride, phase I clinical trials were successfully completed, demonstrating mild nephrotoxicity, fatigue, and bilierubinimia as dose-dependent side effects, which quickly disappeared after the discontinuation of the administration of the drug [12,13].

However, despite promising results in preclinical studies in mice, titanocene dichloride did not show a therapeutic effect during phase II clinical trials at a dose of 240 mg/m^2^ [9]. One of the possible reasons for the failure in clinical trials was the low stability of titanocene dichloride in aqueous media and its rapid hydrolysis, leading to the loss of chloride ligands. The main driving force of hydrolysis is the high oxygenophilicity of titanium and the formation of a stronger Ti-O bond (ΔH_f298_ = 662 kJ/mol) [14] compared to the Ti-Cl bond (ΔH_f298_ = 494 kJ/mol) [14]; therefore, it can be assumed that the replacement of chloride anions in titanocene by oxygen-containing ligands should significantly increase the hydrolytic stability of titanocene derivatives. This is consistent with the data in the literature on attempts to stabilize titanocene in aqueous media by incubating titanocene dichloride in alcoholic solutions (MeOH, EtOH) [15]. It was shown that the resulting compounds have a more pronounced cytotoxic effect than the original titanocene dichloride, and these formulations were also used in preclinical and clinical trials.

Attempts to obtain more stable and more active titanocene derivatives for the treatment of oncological diseases are being undertaken by several scientific groups [16,17,18]; however, a systematic search for new carboxylate-containing titanocene derivatives based on substituted benzoic acids has not yet been carried out.

The factors which determine the cytotoxicity of transition metal coordination compounds (titanocene in particular) are the redox potentials of the complexed metal ion. It was shown that for titanocene derivatives, the key stage determining the effectiveness of the cytotoxic action after the endocytosis of the complex into the cell is the reduction of titanocene to Ti^+2^ derivatives transported to the cell nucleus, with this disrupting the functioning of various metalloenzymes and transcription factors [19].

Furthermore, the redox potential of a coordination compound may affect its stability in biological systems. Thus, by changing the redox potential of titanocene derivatives with oxygen-containing ligands, it is potentially possible to choose the optimal ligands and the values of the redox potential of the coordination compound that correspond to the highest hydrolytic stability in aqueous media and the most efficient release of Ti^+2^ in the cell.

In this article, a series of carboxylate-containing titanocene (IV) derivatives based on benzoic acids containing various donor and acceptor substituents in the benzene ring was synthesized. To obtain target compounds, three alternative synthetic methods were tested, and the optimal method for each specific compound was chosen. The analysis of the influence of the nature and position of the substituent in carboxylate ligands on the potential of the redox transition of the Ti^+4^ ion and the relative stabilities of the complexes in the cyclic redox processes made it possible to formulate conclusions regarding the rational design of new and effective cytotoxic agents based on titanocene (IV).

## 2. Results and Discussion

### 2.1. Synthesis of Titanocene (IV) Dicarboxylates

The most common method for preparing various titanocene derivatives from titanocene dichloride is the nucleophilic displacement of chloride anions with a suitable nucleophilic reagent. Several methods have been described in the literature for the introduction of certain carboxylate anions to titanocene [17,18,20,21], but our attempts to reproduce them only partially lead to satisfactory results because the products obtained by these methods were always isolated from the solution together with resinous impurities, the separation from which requires long-term purification using repeated reprecipitation.

A key feature of the synthesis of carboxylate-substituted derivatives of titanocene is the difficulty of purifying the target product from possible impurities and starting materials. Specifically, the purification of titanocene dicarboxylates by column chromatography on silica gel and aluminum oxide is impossible due to their rapid degradation and the exchange of ligands under the action of the sorbents as well as high-temperature recrystallization, since, when heated above 40–45 °C, the target compounds undergo partial destruction and crystallize with impurities detectable in ^1^H NMR (broadened signals near the titanocene signals).

**Scheme 1:**
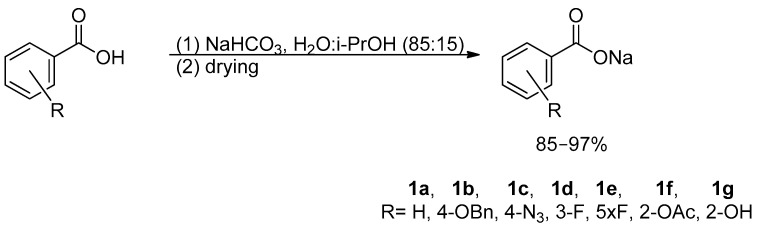
Synthesis of sufficiently pure compounds **1a**–**g**.

In addition, for titanocene dicarboxylate synthesis, it is important to use absolutely dry salts of carboxylic acids, which do not contain even minor impurities of strong and nucleophilic bases, as starting materials. We found that the only way to obtain sufficiently dry and pure sodium salts of carboxylic acids **1a**–**g** was the reaction of the corresponding carboxylic acids with sodium bicarbonate in a mixture of isopropanol and water (1:1), followed by azeotropic drying and the maintaining of samples in a vacuum desiccator over phosphorus pentoxide (Scheme 1). Thus, sodium salts that are sufficiently pure and dry for organometallic reactions of any carboxylic acids strong enough to react with sodium bicarbonate can be successfully prepared by the described method. The use of sodium hydride salts (tested in the framework of this work), sodium ethoxide in ethanol, sodium methoxide in methanol [20], or solutions of sodium hydroxide in water [22] for the preparation of the required salts leads to side processes when sodium salt interacts with titanocene dichloride, even in the case of the preliminary purification of the obtained salts by recrystallization.

Special remarks should be made regarding obtaining the sodium salt of aspirin **1f**. The known methods described in the literature for its preparation in strongly alkaline aqueous media [23,24,25] not only do not make it possible to obtain a high purity compound but often lead to the partial or complete hydrolysis of ester groups. The same conclusions were made by the authors of [26], who experienced similar difficulties in reproducing the methods in the literature for the synthesis of aspirin-containing ruthenocene derivatives.

**Scheme 2:**
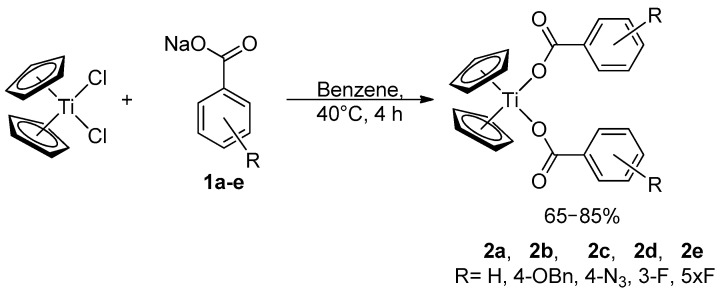
Synthesis of titanocene dicarboxylates **2a**–**e** using sodium carboxylates.

The synthesis of titanocene dicarboxylates **2** was carried out according to Scheme 2. The best yields of target compounds were obtained using absolute benzene as a solvent [20], while our attempts to use methanol, aqueous methanol, chloroform, and acetone did not lead to yields of target compounds greater than 10–15%.

All the tested conditions for the synthesis of the previously described [27] model compound **2a** are given in Table 1. As a result of optimization, we managed to obtain individual compound **2a** with a yield of 87% by direct precipitation from the reaction medium at room temperature. An increase in the reaction temperature or the reaction time leads to a decrease in the yield of titanocene dicarboxylate, since the target compounds partially decompose when their time in solution or heating is prolonged.

Under optimized conditions, other titanocene dicarboxylates **2b**–**e** were also obtained in high yields (Scheme 2). However, compound **2f**, containing aspirin fragments, was isolated from the reaction mixture only in trace amounts and contained inseparable impurities of certain by-products with titanocene fragments.

**Scheme 3:**
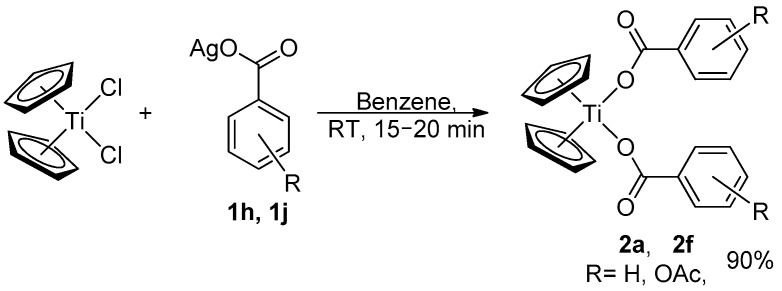
Synthesis of titanocene dicarboxylates **2a**, **2f** using silver carboxylates.

To solve this problem, an additional variation of the conditions was carried out, namely, the search for other counterions for the aspirin salt were made (Scheme 3). Using compound **2a** as a model compound, the procedure for the synthesis of the target titanocene dicarboxylate based on more active silver salts of carboxylic acids was optimized (see Supplementary Materials).

The precipitation of silver chloride during the reaction quickly shifts the equilibrium towards the formation of target products. It was shown that in the case of compound **2a**, complete conversion is achieved in just 15 min at room temperature (instead of 4 h at 40 °C for a similar reaction with sodium benzoate). For the compound **2f**, the replacement of sodium aspirinate **1f** with silver aspirinate **1i** also accelerated the reaction and increased the conversion of the starting compounds, but the reaction product was always contaminated with inseparable impurities containing titanocene fragments. An additional disadvantage of this method is the need to carry out all operations with silver salts in the dark or under low artificial light due to their low stability in sunlight.

**Scheme 4:**
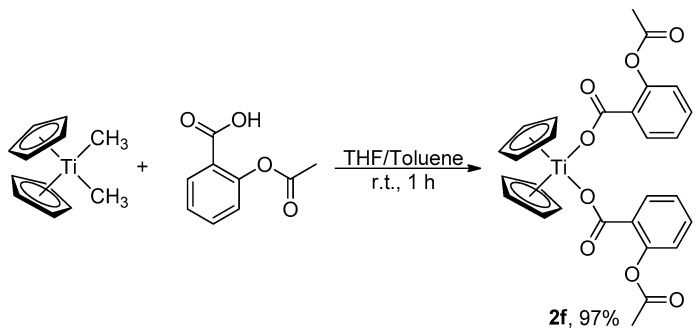
Synthesis of compound **2f** from dimethyltitanocene and aspirin.

We also tested a third alternative method for titanocene dicarboxylate synthesis, considering that the aspirin salts may undergo partial deacetylation in solution, which leads to the formation of mixtures of structurally similar coordination compounds with one or two acyl groups. In this method, to obtain a pure compound, **2f**, we used the aspirin on its own and not in the form of a carboxylate salt. Using dimethyltitanocene, which is capable of reacting with the carboxyl group of aspirin, as a starting Ti (IV) derivative, it was possible to obtain the target compound **2f** in a high yield and not contaminated by side products (Scheme 4). This method allows the reaction to be carried out in a moderately acidic medium and does not require the use of basic carboxylic acid salts, which, most likely, are the cause of the formation of by-products.

All the obtained titanocene dicarboxylates **2a**–**f** were characterized by ^1^H, ^13^C, ^19^F (for fluorine-containing compounds) NMR spectroscopy as well as by elemental analysis.

Thus, we tested and optimized three methods for the synthesis of titanocene dicarboxylates. The first method, which consists of the interaction between titanocene dichloride and sodium salts of carboxylic acids, is the simplest to carry out and is suitable for introducing into the structure of titanocene the salts of most carboxylic acids strong enough to obtain a sodium salt with sodium bicarbonate. The disadvantage of this technique is that the reaction must be carried out at an elevated temperature, which is not suitable for certain labile carboxylic acids. The second synthetic method consists of the interaction between titanocene dichloride and silver salts of carboxylic acids. Its advantages are high yields of the target compounds, the ability to carry out reactions at room temperature, and short reaction times. However, the preparation of silver salts is a laborious procedure, and all reactions involving them must be carried out in the dark. The third method is the interaction between dimethyltitanocene and carboxylic acids. Its advantage is mild reaction conditions (room temperature), and this method is suitable for the preparation of titanocene dicarboxylates based on carboxylic acids that are labile or unstable when heated. However, dimethyltitanocene is synthesized by a complex two-step procedure [28], and it is unstable during long-term storage both in solid form and in solution.

**Scheme 5:**
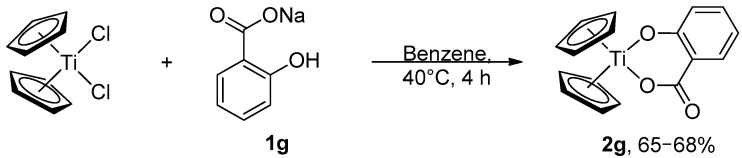
Synthesis of compound **2g** with bidentate binding mode of the salicylic acid.

Compound **2g**, containing a salicylic acid residue as a bidentate ligand, can be obtained under the conditions described in this work (sodium salt method) in 60% yield or according to the method in [29] in 68% yield (Scheme 5).

The bidentate nature of the ligand binding in the titanocene **2g** with the formation of a six-membered chelate cycle turns out to be so beneficial that it is not possible to obtain titanocene dicarboxylate based on salicylic acid under any of the tested conditions and by none of the methods in the literature [21,22,30,31,32]. A consequence of the increased stability of compound **2g** is the fact that this is the only titanocene derivative in this work for which it was possible to obtain a molecular ion in the HRMS spectrum (see Supplementary Materials). Compounds **2a**–**f**, meanwhile, underwent degradation with the loss of both ligands under ESI conditions, which did not allow us to register informative mass spectra.

### 2.2. Molecular Structure Oftitanocene (IV) Dicarboxylates ***2a**, **2e**, **2g***

To obtain single crystals suitable for X-ray diffraction analysis for titanocene derivatives, in this work, crystallization from a solution using the gas-phase diffusion of an antisolvent (see Section 3.4) was used for the first time, which makes it possible to obtain much better and larger crystals compared to classical crystallization methods by cooling the solution or slow evaporation solvent [17,20].

**Figure 1:**
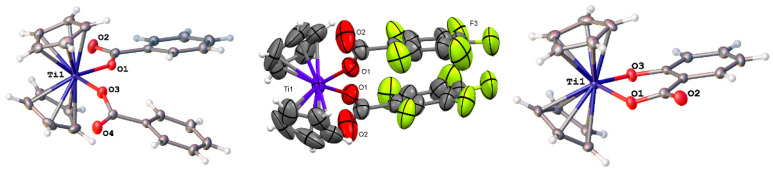
Molecular structure of titanocene (IV) dicarboxylates **2a** (**left**), **2e** (**middle**), and **2g** (**right**) in representation of atoms by thermal ellipsoids (*p* = 50%).

The molecular structures of coordination compounds of both structural classes containing two identical ligands or one bidentate ligand are shown in Figure 1. Selected bond lengths and angles important for understanding the geometry of molecules **2a**–**g** in crystals are given in Table 2.

The Cambridge Structural Database contains the crystallographic and geometrical parameters of more than twenty structures with the general formula Cp_2_Ti(O_2_CR)_2_. An analysis of these data (Ti-C, Ti-O bond lengths, and Cp-Ti-Cp angle), characterizing the interaction of the cation and the nearest atoms, allows us to conclude that the corresponding parameters in different molecules do not differ significantly and that they are equal to the values found in the structures presented by us in this paper (see Figure 1 and references in this section).

The main differences are in the spatial arrangement of the radicals R,R. This arrangement is determined by many factors such as the steric interaction R, i.e., whether they are among themselves within one molecule or there is evidence of the pi-conjugation of the carboxylate group with R and intermolecular interactions, including hydrogen bonds C-H...O (2).

If R-Ph, then the torsion angle O-C-C1(Ph)-C2(Ph) (C1 and C2 are atoms of the phenyl ring; the carboxylate group is connected with the first, the next Ph atom and the hydrogen atom.) varies within 15° (20° according to [20]). The ability to rotate Ph around the C–C1 bond is a factor that makes it possible to reduce the steric repulsion between R,R of the same molecule. On the contrary, if this possibility is made more energetically costly (for example, in the C2 position, the hydrogen is replaced by an oxy group, thereby organizing an intramolecular hydrogen bond in the O-C-C1-C2(OH) fragment), then to overcome the steric repulsion between the RR radicals, other methods are required.

The XRD have revealed that a single crystal of **2a,** obtained by our method, crystallizes at 110 K in the P2_1_2_1_2_1_ space group and is almost identical to data in the literature (at 155 and 115 K) related to an orthorhombic polymorph of **2a** (Table S1) [27,33].

In contrast, for **2g**, the crystal structure at 100 K differ from those at room temperature [34]. More precisely, cooling the crystal to 110 K results in a threefold increase in the volume of the unit cell. The spatial group P2_1_/c and two-unit cell parameters (a and c) remain unchanged, while the parameter b increases threefold and the number of independent molecules increases from one to three (Z = 12, Z′ = 3) (Table S1). Three independent molecules differ slightly via the conformation of 6-membered metallocycle. The observed difference in two crystal strictures with Z′ = 3 and Z′ = 1 is more likely the consequence of phase transition.

Finally, the crystal structure of **2e** is novel. The complex crystallizes in the centrosymmetric space group and occupies the 2-fold axis passing through the titanium. The oxygen atoms bonded to titanium are located in the equatorial plane. In the structure of the 2e molecule presented in this work, the OOCPh conjugation is completely eliminated by the repulsion of the oxygen atom O2 and the fluorine atom F5 (respectively, O2i…F5i); the torsion angle between the carboxylate group and Ph is approximately 50 degrees, which makes it possible to reduce the steric repulsion of pentafluorophenyl rings, with their almost parallel arrangement.

**Figure 2:**
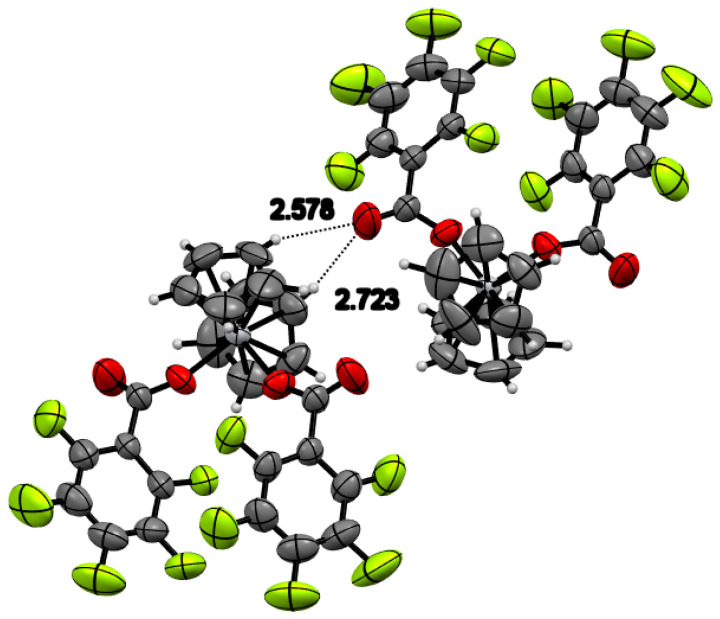
Molecular structure of titanocene (IV) dicarboxylate **2e** with O…H contacts.

Regarding the intermolecular hydrogen bonds, it should be noted that the bonds characteristic [20] of these compounds are those in which the hydrogen atoms of two Cp anions of one molecule interact with the uncoordinated oxygen atom of the carboxylate group (Figure 2) of the neighboring one. Bonds O–H are between 2.6–2.7 Å long.

### 2.3. Electrochemistry of Titanocene (IV) Dicarboxylates

Ti (IV) derivatives are oxidizing agents, and the mechanism of the cytotoxic action of titanocenes includes the stage of its stepwise reduction to Ti^+2^ intermediates [19]. Since the many possible mechanisms of the cytotoxic action of transition metal coordination compounds are associated with the occurrence of redox reactions inside the cell, the titanocene **2a**–**g** carboxylates obtained in this work were studied by cyclic voltammetry.

It was shown that, as expected, the reduction potentials of Ti (IV) for all compounds depend on the structure of the carboxylate-containing ligand, in particular, the presence of donor and acceptor substituents in the ligand benzene ring (Table 3).

As expected, the introduction of donor substituents into the benzene ring of the carboxylate ligand shifts the reduction potential from −0.85 V (**2a**) to −0.88 V (**2b**) and −0.90 V for most donating *ortho* phenolate containing bidentate ligand **2g**. The introduction of acceptor substituents, on the contrary, shifts the reduction potential up to −0.57 V (**2e**). Substituents with moderate acceptor properties such as 3-F (**2d**) and 4-N_3_ (**2c**) occupy an intermediate position, shifting the potential to the values of −0.79 V and −0.78 V, respectively. The substituent in the *para* position had the greatest influence.

**Figure 3:**
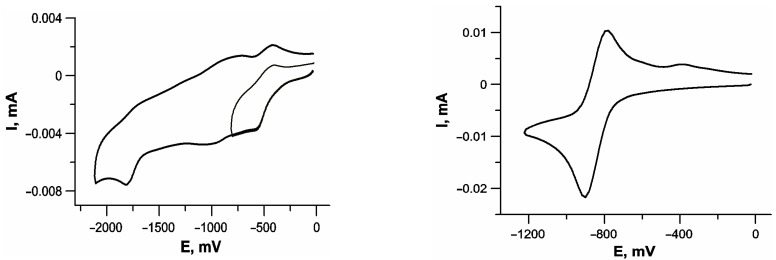
CV-curves of compounds **2e** (**left**) and **2g** (**right**).

**Scheme 6:**
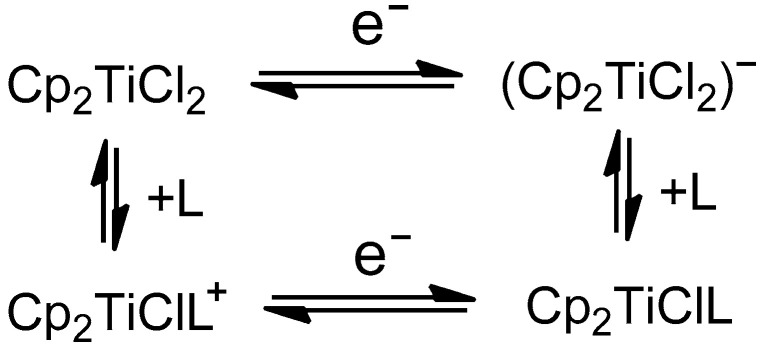
Possible pathways of titanocene dichloride single electron reduction [35].

The analysis of the shape of the CV curves shows the absence of reversibility during re-oxidation after reduction (Figure 3), which allows for the conclusion that in the course of redox reactions in polar solvent (DMF), carboxylate-substituted derivatives of titanocene, as well as titanocene dichloride itself (Scheme 6) [35], undergo stepwise reduction with the loss of one of the two ligands dissociating from the Ti ion. In the course of the oxidation of the obtained particle, the vacant position appears to be occupied by the solvent molecule (DMF) and not by the initial ligand.

The only compound that is reversibly reduced and oxidized is compound **2g**. This may be explained by the fact that the bidentate binding of the ligand in this complex does not allow it to completely dissociate from the molecule of the coordination compound during the one-electron reduction process. Carrying out several redox cycles does not lead to a potential shift and, therefore, does not lead to a change in the structure of compound **2g**.

Thus, according to the electrochemical studies, it can be concluded that the optimal ligands for obtaining biologically active titanocene derivatives are acceptor carboxylate ligands and ligands bidentately bound to the metal atom. Firstly, titanocenes with such carboxylate ligands can be more easily activated to cytotoxically active titanium(II) derivatives by intracellular reducing agents, such as glutathione and thioredoxin, etc., in which the redox transition occurs at ~−0.24–−0.32 V [36,37]. Secondly, the bidentate binding of the ligand allows the ligand not to dissociate from the titanocene molecule during the first one-electron reduction, affecting not only the Ti (IV)/Ti (III) transition potential but also the potential of the subsequent Ti (III)/Ti (II) reduction. Thirdly, due to the reversibility of the reduction, it is possible for them to carry out a cyclic redox process that contributes to cytotoxicity due to the generation of ROS [38,39]. Acceptor substituents reduce the reduction potential of titanocene, and the bidentate nature of binding increases the stability of the complex. Therefore, the combination of these factors must be taken into account in the design of carboxylate-containing ligands for titanocene based drug candidates.

### 2.4. Cytotoxicity of Titanocene (IV) Dicarboxylates

**Figure 4:**
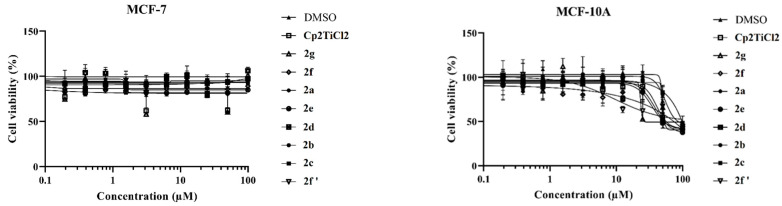
Cell viability curves in presence of titanocene dicarboxylates **2a**–**g** for MCF7 (**left**) and MCF7-10A (**right**).

Breast cancer cells have been used for testing the cytotoxicity of Ti organic complexes [16,18]. We therefore examined the ability of our newly synthesized titanocene dicarboxylates to kill human malignant (MCF-7) or non-malignant (MCF-10A) cell lines. None of the tested compounds were cytotoxic at concentrations <100 µM within 72 h of continuous exposure (Figure 4). These results are in line with the limited cytotoxicity reported for this particular class. Nevertheless, we tend to interpret these data as an opportunity to design chemically stable Ti organic derivatives conjugated with conventional or novel chemotherapeutics.

The low toxicity of all obtained compounds prompted us to investigate their stability in aqueous media in order to exclude the factor of an insufficient lifetime in water from the possible causes of low cytotoxicity.

### 2.5. Stability in Aqueous Media

To study their stability in aqueous media, compounds **2b** (the most electron-donating ligand), **2e** (the most electron-withdrawing ligand), and **2g** (a chelating bidentate ligand) were chosen. Thus, representatives of all the compounds obtained in the work were studied (See Section 3 for experimental details).

**Figure 5:**
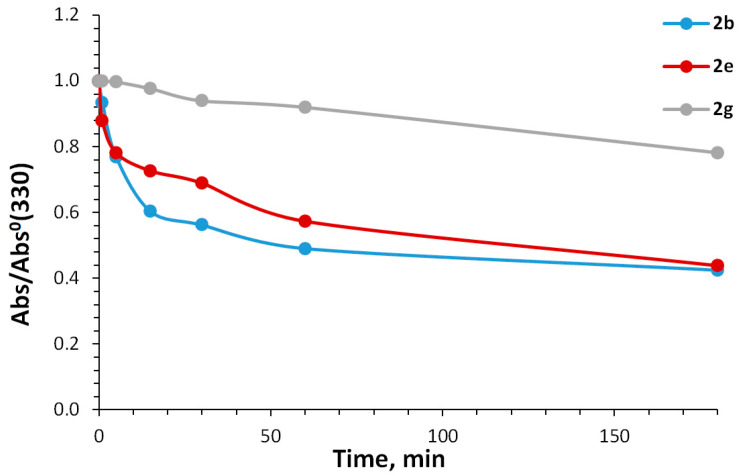
Kinetic curves of hydrolysis of titanocene derivatives **2b**, **2e**, **2g**, obtained from the value of optical absorption at a wavelength of 330 nm.

The decrease in the concentration of the coordination compound was estimated from the decrease in the optical absorption at a wavelength of 330 nm. We considered it inappropriate to use the optical absorption maximum in the range of 230–300 nm for similar purposes because the absorption of the initial coordination compound, free carboxylate-containing ligands, and cyclopentadiene may overlap in this region. The hydrolysis kinetic curves obtained by this method are shown in Figure 5.

The kinetic curve for compound **2g** can be attributed to first order kinetics. Most likely, the reaction consists of the transition of the chelate molecule to the monohydrate with the retention of the ligand on the titanium atom. Hydrolysis proceeds slowly, which is consistent with other data on the high stability of this compound. The kinetic curves for compounds **2b** and **2e** cannot be reduced to integer kinetic orders or are straightened in semilogarithmic coordinates. Most likely, in this case, a more complex kinetic scheme is observed, with the hydrolysis of the first and then the second carboxylate-containing ligand and their dissociation from titanium. It can be seen from the presented curves that compound **2e,** containing acceptor ligands, is hydrolyzed more slowly than compound **2b**, with donor ligands. These data, as well as the data of electrochemical studies, make it possible to name acceptor ligands and ligands that are bidentately bound to titanium as the most promising for the preparation of biologically active titanocene derivatives.

It was not possible to measure the hydrolysis kinetics of the starting titanocene dichloride under similar conditions, which indicates its lower stability in aqueous media compared to the carboxylate-containing analogues synthesized in this work. The results on the extremely low stability of titanocene dichloride in aqueous media are confirmed by data in the literature [40].

## 3. Materials and Methods

### 3.1. General Information

All reagents were obtained from Sigma Aldrich (unless specified otherwise) and used without further purification. NMR measurements were carried out on a Bruker-Avance 400 MHz spectrometer in DMSO-d6 and CDCl_3_ using the solvent residual peak as an internal reference. 

**Elemental analysis** of the synthesized compounds was made on the Vario MICRO Cube by ELEMENTAR.

**IR-spectra** of compounds were recorded on an IR spectrometer with Fourier transform TermoNicolet ISFT-IR (USA) by frustrated total internal reflection method (FTIR).

**Electrospray ionization high-resolution mass spectra** were recorded in positive ion mode on a TripleTOF 5600+ quadrupole time-of-flight mass spectrometer (ABSciex, Concord, Vaughan, ON, Canada) equipped with a DuoSpray ion source. The following MS parameters were applied: capillary voltage 5.5 kV; nebulizing and curtain gas pressure—15 and 25 psi, respectively; ion source temperature—ambient; declustering potential 20 V; *m*/*z* range 100–1200. Elemental compositions of the detected ions were determined based on accurate masses and isotopic distributions using Formula Finder software (ABSciex, Concord, ON, Canada). The maximum allowed deviation of the experimental molecular mass from the calculated one was 5 ppm.

**Cyclic Voltammetry** experiments were conducted using an IPC Pro M potentiostat (Russia) with cyclic voltammetry (CV) and rotating disk electrode (RDE) techniques. Glass-carbon (d = 2 mm) disks were used as the working electrodes, 0.05 M Bu_4_NClO_4_ solution in DMF served as the supporting electrolyte, and Ag/AgCl/KCl (sat.) was used as the reference electrode. The potential scan rates were 100 mV/s. Samples were dissolved in the predeaerated solvent.

**Stability in aqueous media** was studied by recording the full optical absorption spectra of solutions of the studied titanocene derivatives with an initial concentration of 0.4 mM in the solvent system (10% DMSO 90% water) at different time intervals. The change in the concentration of the studied compound was evaluated by the decrease in optical absorption at a wavelength of 330 nm. Full absorption spectra of studied compounds are presented as Figure S7 in the Supplementary Materials.

**Titanocene dichloride** was synthesized by a previously reported procedure with minor alterations [41]. ^1^H NMR (CDCl_3_, 400 MHz, δ ppm): 6.59 (s, 10H, Cp). ^13^C NMR (CDCl_3_, 101 MHz, δ ppm): 119.8. Elemental analysis: calculated (%) for C_10_H_10_Cl_2_Ti, 48.24, H, 4.05; found C 49.13, H 4.28, N < 0.03, S < 0.03.

**Solution of dimethyltitanocene** was obtained by using a previously reported procedure [28] with use of methyl magnesium iodide instead of methyl magnesium chloride. ^1^H NMR (CDCl_3_, 400 MHz, δ ppm): 6.04 (s, 10H, Cp), −0.14 (s, 6H, 2xCH_3_).

**Pentafluorobenzoic acid** was synthesized by using pentafluorobenzoic acid, which was obtained by passing a stream of air through a solution of pentafluorobenzaldehyde in dry acetone for 8 h. The acid was isolated in quantitative yield as white crystals and used without further purification [42].

**4-azidobenzoic acid** was obtained via the diazotization of 4-aminobenzoic acid under standard conditions. A total of 0.75 mL of concentrated HCl was added dropwise to a suspension of 4-aminobenzoic acid (400 mg, 2.9 mmol) in 4 mL of water in a laboratory beaker, and the resulting solution was cooled with ice to 0 °C, with sodium nitrite (212 mg, 3.1 mmol) being added in small portions with stirring. After two hours at room temperature, the reaction mixture was extracted with ethyl acetate, the organic layer was washed with water and brine, dried over anhydrous sodium sulfate, and dried on a rotary evaporator. 4-Azidobenzoic acid was obtained as a light-yellow powder. Yield 420 mg, 88%. ^1^H NMR (DMSO-d6, 400 MHz, δ ppm): 12.97 (br. s., 1H, -COOH), 7.96 (m, 2H, -CH-Ar), 7.20 (m, 2H, -CH-Ar). IR: 2102, 1673, 1600, 1423, 1282, 1177, 934, 857, 766, 556. IR: The appearance of a strong absorption band at 2102 cm^−1^ and the disappearance of absorption bands at 3460 and 3360 cm^−1^ (Figure S1) in comparison with the spectrum of the starting compound indicated the complete conversion of the amino group to azide.

### 3.2. Synthesis of Carboxylic Acids Sodium Salts

**General procedure:** A mixture of dry carboxylic acid (2.5 mmol) and sodium bicarbonate (2.5 mmol) was poured with a mixture of water and isopropyl alcohol 1:1 (5 mL), with the reaction mixture then being stirred in an open flask at room temperature until the end of gas evolution. The resulting mixture (pH = 7) was evaporated to dryness on a rotary evaporator at a water bath temperature of 40 °C. The removal of traces of water was achieved by double azeotropic drying with benzene and keeping the resulting powder of the sodium salt of the carboxylic acid in a vacuum desiccator over phosphorus pentoxide for 12 h.

Sodium benzoate **1a**: was obtained from local suppliers, dried, and used without characterization.

Sodium 4-benzoxybenzoate **1b**: From 4-benzoxybenzoic acid (570 mg, 2.5 mmol) and sodium bicarbonate (210 mg, 2.5 mmol) **1b** was obtained as a white crystalline precipitate. Yield 594 mg, 95%. ^1^H NMR (DMSO-d6, 400 MHz, δ ppm): 7.82 (m, J = 8.06 Hz, 2H, -CHα-Ar), 7.31–7.47 (m, 5H, -Ph), 6.93 (m, J = 8.11 Hz, 2H, -CHβ-Ar), 5.11 (s, 2H, -CH_2_-).

Sodium 4-azidobenzoate **1c**: **1c** was obtained as a white crystalline precipitate from 4-azidobenzoic acid (407 mg, 2.5 mmol) and sodium bicarbonate (210 mg, 2.5 mmol). Yield 416 mg, 90%. ^1^H NMR (D_2_O, 400 MHz, δ ppm): 7.78 (d, J = 8.66 Hz, 2H, -CHα-Ar), 7.00 (d, J = 8.66 Hz, 2H, -CHβ-Ar).

Sodium 3-fluorobenzoate **1d**: **1d** was obtained as a white crystalline precipitate from 3-fluorobenzoic acid (350 mg, 2.5 mmol) and sodium bicarbonate (210 mg, 2.5 mmol). Yield 393 mg, 97%. ^1^H NMR (DMSO-d6, 400 MHz, δ ppm): 7.70 (d, J = 7.56 Hz, 1H, -CHα-Ar), 7.57 (ddd, J = 10.29, 2.67, 1.01 Hz, 1H, -CHα’-Ar), 7.26–7.37 (m, 1H-CHγ-Ar), 7.07–7.15 (m, 1H-CHβ-Ar).

Sodium pentafluoro benzoate **1e**: **1e** was obtained as a white crystalline precipitate from pentafluorobenzoic acid (530 mg, 2.5 mmol) and sodium bicarbonate (210 mg, 2.5 mmol). Yield 556 mg, 95%. ^1^H NMR (D_2_O, 400 MHz, δ ppm): empty. ^19^F NMR (D_2_O, 376.5 MHz, δ ppm): −144.25 (m, 2F), −155.76 (m, 1F), −161.97 (m, 2F).

Sodium acetylsalicylate **1f**: **1f** was obtained as a white crystalline precipitate from Aspirin (540 mg, 2.5 mmol) and sodium bicarbonate (210 mg, 2.5 mmol). Yield 353 mg, 70%. ^1^H NMR (D_2_O, 400 MHz, δ ppm): 7.61 (dd, J = 7.67, 1.59 Hz, 1H, -CHα-Ar), 7.40–7.47 (m, 1H, -CHβ-Ar), 7.26–7.32 (m, 1H, -CHβ’-Ar), 7.08 (m, 1H, -CHγ-Ar), 2.26 (s, 3H, CH_3_).

Sodium salicylate **1g**: **1g** was obtained as a white crystalline precipitate from salicylic acid (345 mg, 2.5 mmol) and sodium bicarbonate (210 mg, 2.5 mmol). Yield 340 mg, 85%. ^1^H NMR (DMSO-d6, 400 MHz, δ ppm): 7.60–7.71 (m, 1H, -CHα-Ar), 7.08–7.17 (m, 1H, -CHγ-Ar), 6.53–6.66 (m, 2H, -CH-Ar-β,β′).

### 3.3. Synthesis of Titanocene (IV) Dicarboxylates

**General procedure:** a suspension of titanocene dichloride (0.2 mmol) in absolute benzene (4 mL) was added to a suspension of the sodium salt of a carboxylic acid (0.4 mmol) in absolute benzene (2 mL) with stirring. The flask was placed on the Schlenk line, the solution was degassed, and the system was filled with argon three times, after which the reaction mixture was stirred while heated on a water bath at a temperature of 40 °C for 4 h. The resulting light orange solution was filtered off from the NaCl precipitate, evaporated on a rotary evaporator, and reprecipitated from the methylene chloride:petroleum ether system. The target compounds are stable when stored on air in a refrigerator.

If silver salts were used instead of sodium salts for the reaction, the reactions were carried out at night under artificial lighting at room temperature, and the reaction time was reduced from 4 h to 15–20 min.

Synthesis of bis(η^5^-cyclopentadienyl)bis(benzoato)titanium(IV) **2a**: *Method A*: **2a** was obtained as a light orange precipitate from sodium benzoate **1a** (57.6 mg, 0.4 mmol) and titanocene dichloride (50 mg, 0.2 mmol). Yield 73 mg, 87%. ^1^H NMR (CDCl_3_, 400 MHz, δ ppm): 8.02–8.09 (m, 4H, 4x-CHα,α′-Ar), 7.52–7.57 (m, 2H, 2x-CHγ-Ar), 7.46 (t, J = 7.45 Hz, 4H, 4x-CHβ,β′-Ar), 6.60–6.69 (m, 10H, 2xCp). ^13^C NMR (CDCl_3_, 101 MHz, δ ppm) 172.1, 133.7, 131.8, 129.9, 128.2, 118.5, 118.5. *Method B*: **2a** was obtained as a light orange precipitate from silver benzoate **1h** (183 mg, 0.8 mmol) and titancene dichloride (100 mg, 0.4 mmol). Yield 152 mg, 90%. ^1^H NMR (CDCl_3_, 400 MHz, δ ppm): 8.03–8.08 (m, 4H, 4x-CHα,α′-Ar), 7.52–7.57 (m, 2H, 2x-CHγ-Ar), 7.46 (t, J = 7.48 Hz, 4H, 4x-CHβ,β′-Ar), 6.62–6.67 (m, 10H, 2xCp). Elemental analysis: calculated (%) for C_24_H_20_O_4_Ti C, 68.59; H, 4.80; found C 68.18, H 4.53, N 0.10, S < 0.03.

Synthesis of bis(η^5^-cyclopentadienyl)bis(4-benzoxybenzoato)titanium(IV) **2b**: **2b** was obtained as a light orange precipitate from sodium 4-benzoxybenzoate **1b** (100 mg, 0.4 mmol) and titanocene dichloride (50 mg, 0.2 mmol). Yield 101 mg, 80%. ^1^H NMR (CDCl_3_, 400 MHz, δ ppm): 7.99 (d, J = 8.77 Hz, 4H, 4x-CHα,α′-Ar), 7.39–7.49 (m, 10H, 2xPh), 7.01 (d, J = 8.82 Hz, 4H, 4x-CHβ,β′-Ar), 6.57–6.66 (m, 10H, 2xCp), 5.15 (s, 4H., 2x-CH_2_-O-). ^13^C NMR (CDCl_3_, 101 MHz, δ ppm): 172.02, 161.67, 136.42, 131.85, 128.65, 128.14, 127.46, 126.46, 118.41, 114.27, 70.05. Elemental analysis: calculated (%) for C_38_H_32_O_6_Ti*HCl, 68.22; H, 4.97; found C 68.63, H 4.73, N 0.05, S < 0.03.

Synthesis of bis(η^5^-cyclopentadienyl)bis(4-azidobenzoato)titanium(IV) **2c**: **2c** was obtained as a light orange precipitate from sodium 4-azidobenzoate **1c** (74 mg, 0.4 mmol) and titanocene dichloride (50 mg, 0.2 mmol). Yield 65 mg, 65%. ^1^H NMR (CDCl_3_, 400 MHz, δ ppm): 7.96–8.05 (m, 4H, 4x-CHα,α′-Ar), 7.06–7.10 (m, 4H, 4x-CHβ,β′-Ar), 6.60–6.66 (m, 10H, 2xCp). ^13^C NMR (CDCl_3_, 101 MHz, δ ppm): 171.33, 143.62, 131.92, 131.62, 130.30, 118.67. Elemental analysis: calculated (%) for C_24_H_18_N_6_O4Ti C, 57.39; H, 3.61; N, 16.73; found C 57.70, H 3.79, N 17.52, S < 0.03.

Synthesis of bis(η^5^-cyclopentadienyl)bis(3-fluorobenzoato)titanium(IV) **2d**: **2d** was obtained as a light orange precipitate from sodium 3-fluorobenzoate **1d** (65 mg, 0.4 mmol) and titanocene dichloride (50 mg, 0.2 mmol). Yield 73 mg, 80%. ^1^H NMR (CDCl_3_, 400 MHz, δ ppm): 7.83 (d, J = 7.67 Hz, 2H, 2x-CHα-Ar), 7.71 (d, J = 9.37 Hz, 2H, 2x-CHα′-Ar), 7.40–7.47 (m, 2H,2x-CHβ-Ar), 7.21–7.26 (m, 2H, 2x-CHγ-Ar), 6.65 (s, 10H, 2xCp). ^13^C NMR (CDCl_3_, 101 MHz, δ ppm) 171.3, 143.6, 131.9, 131.6, 130.3, 118.7. Elemental analysis: calculated (%) for C_24_H_18_F_2_O4Ti C, 63.18; H, 3.98; found C 62.59, H 4.05, N 0.08, S < 0.03.

Synthesis of bis(η^5^-cyclopentadienyl)bis(pentafluorobenzoato)titanium(IV) **2e**: **2e** was obtained as a light orange crystalline precipitate from sodium pentafluorobenzoate **1e** (93.6 mg, 0.4 mmol) and titanocene dichloride (50 mg, 0.2 mmol). Yield 102 mg, 85%. ^1^H NMR (CDCl_3_, 400 MHz, δ ppm): 6.69 (s, 10H, 2xCp). ^19^F NMR (CDCl_3_, 376.5 MHz, δ ppm): −141.45 (m, 2F), −152.56 (m, 1F), −161.36 (m, 2F).

Synthesis of bis(η^5^-cyclopentadienyl)bis(2-acetoxybenzoato)titanium(IV) **2f**: *Method A*: **2f** was obtained as a light orange precipitate from sodium acetylsalicylate **1f** (81 mg, 0.4 mmol) and titanocene dichloride (50 mg, 0.2 mmol). Yield: traces. *Method B*: **2f** was obtained as a light orange precipitate (with inseparable impurities) from silver acetylsalicylate **1i** (230 mg, 0.8 mmol) and titanocene dichloride (100 mg, 0.4 mmol) after 15 min. Yield:193 mg, 90% of impure compound. ^1^H NMR (CDCl_3_, 400 MHz, δ ppm): 7.91 (dd, J = 7.73, 1.74 Hz, 2H, -CHα-Ar), 7.49–7.54 (m, 2H, -CHβ-Ar), 7.24 (td, J = 7.58, 1.22 Hz, 2H, -CHβ-Ar), 7.11 (dd, J = 8.01, 1.16 Hz, 2H, -CHγ-Ar), 6.62 (s, 10H, 2xCp), 2.37 (br.s., 6H, 2xCH_3_); Impurity 6.58 (m), 6.48 (m). *Method C*: A solution of aspirin (86.5 mg, 0.48 mmol) in 2 mL of tetrahydrofuran was added dropwise to a toluene-tetrahydrofuran solution of dimethyltitanocene containing (50 mg, 0.24 mmol) of dimethyltitanocene at room temperature in a stream of argon. The reaction mixture was stirred until the end of the evolution of methane and then for another 45 min. The solvents were removed on a rotary evaporator, and the solid residue was reprecipitated from the methylene chloride:petroleum ether system. **2f** was obtained as a light orange precipitate. Yield 125 mg, 97%. ^1^H NMR (CDCl_3_, 400 MHz, δ ppm): 7.90 (dd, J = 7.73, 1.64 Hz, 2H, -CHα-Ar), 7.51 (td, J = 7.69, 1.67 Hz, 2H, -CHβ-Ar), 7.20–7.26 (m, 2H, -CHβ-Ar), 7.10 (dd, J = 7.97, 0.74 Hz, 2H, -CHγ-Ar), 6.55–6.65 (s, 10H, 2xCp), 2.35–2.39 (br.s., 6H, 2xCH_3_). ^13^C NMR (CDCl_3_, 101 MHz, δ ppm): 170.1, 169.9, 150.0, 132.5, 132.2, 126.8, 125.7, 123.2, 118.8, 21.4. Elemental analysis: calculated (%) for C_28_H_24_O_8_Ti C, 62.70; H, 4.51; found C 62.75, H 4.25, N 0.09, S < 0.03.

Synthesis of bis(η^5^-cyclopentadienyl)salicylatotitanium(IV) **2g**: *Method A*: **2g** was obtained as a dark purple crystalline precipitate from sodium salycilate **1g** (32 mg, 0.2 mmol) and titanocene dichloride (50 mg, 0.2 mmol). Yield 40 mg, 60%. *Method B*: A solution of titanocene dichloride (498 mg, 2 mmol) and salicylic acid (303 mg, 2.2 mmol) in 30 mL of chloroform was added dropwise to 10 mL of water with stirring and was stirred at 30 °C for 2 h. The organic layer was separated, washed with cold water and brine, and then dried over anhydrous sodium sulfate. The resulting dark purple solution was evaporated on a rotary evaporator, and the dark residue was reprecipitated from the methylene chloride:petroleum ether 1:2 system and washed with a small amount of diethyl ether on the filter [29]. Yield 450 mg, 68%. ^1^H NMR (CDCl_3_, 400 MHz, δ ppm):8.19 (dd, J = 7.87, 1.56 Hz, 1H), 7.40 (m, 1H, -CHα-Ar), 6.90 (m, 1H, -CHβ-Ar), 6.57–6.68 (m, 1H, -CHγ-Ar), 6.42 (s, 10H, 2xCp). ^13^C NMR (CDCl_3_, 101 MHz, δ ppm): 170.4, 167.4, 133.4, 132.3, 120.5, 119.4, 118.7. Elemental analysis: calculated (%) for C_17_H_14_O_3_Ti C, 64.99; H, 4.49; found C 64.91, H 4.37, N 0.07, S < 0.03.

### 3.4. Obtaining Crystals for X-ray Diffraction Analysis

A solution of 20 mg of titanocene dicarboxylate in 500 μL of methylene chloride was placed in an open 4 mL vial, after which the vial was placed on the bottom of the 20 mL vial containing 4 mL of pentane, and the outer vial was tightly closed. Crystals suitable for X-ray diffraction were formed after the diffusion of pentane vapor into methylene chloride after 24–48 h.

### 3.5. Cell Lines and Cytotoxicity Assays

The MCF-7 breast carcinoma cell line (Institute of Cytology, Russian Academy of Sciences, Saint-Petersburg, Russia) was cultured in Dulbecco’s modified Eagle’s medium (DMEM; Biolot, St. Petersburg, Russia) supplemented with 10% fetal bovine serum (BioWest, Nuaillé, France), 10 µg/mL human recombinant insulin, and 50 µg/mL gentamicin. The MCF-10A non-malignant breast epithelial (American Type Culture Collection, Manassas, VA, USA) cell line was propagated in DMEM/F12 (Biolot, Russia) supplemented with 5% heat-inactivated donor horse serum (Biolot), human recombinant insulin (10 µg/mL), epidermal growth factor (20 ng/mL), 0.5 mg/mL hydrocortisone, and 50 µg/mL gentamicin. Cell lines were cultured at 37 °C in a 5% CO_2_ humidified atmosphere. Cells were routinely tested for mycoplasma contamination. Cells in the logarithmic phase of growth were used in the experiments. The cytotoxicity of newly synthesized compounds was evaluated in a MTT test as described in [43].

## 4. Conclusions

In this work, three alternative methods for the synthesis of symmetrical dicarboxylate-substituted titanocene derivatives were compared and optimized, such as the interaction of titanocene dichloride with (1) sodium or (2) silver salts of substituted benzoic acids as well as the (3) interaction of dimethyltitanocene with benzoic acids themselves. It was shown that in the case of stable benzoic acids, the most simple and convenient method for titanocene dicarboxylate synthesis is the interaction of sodium salts of carboxylic acids with titanocene dichloride, which provides high yields of the individual target products. For carboxylic acids with low nucleophilicity of the corresponding sodium salts or low thermal stability, it is possible to increase the reaction rate at room temperature and obtain a high yield of the target product using silver carboxylates that are more active in the reactions with titanocene dichloride but more difficult to obtain and store. In the case of labile benzoic acids (for example, aspirin, which can be partially deacetylated at 40 °C in benzene under the action of another molecule of sodium aspirinate) or acids available only in small quantities (carrying out an additional synthetic step for obtaining and purifying the salt is difficult), the optimal method of synthesis is the direct reaction of dimethyltitanocene and carboxylic acid at room temperature.

The technique involving the gas-phase diffusion of an antisolvent, applied to the carboxylate-substituted titanocene derivatives for the first time, showed excellent results and made it possible to obtain high-purity single crystals from all tested compounds.

It was also demonstrated on a series of dicarboxylate-substituted titanocene derivatives that the introduction of donor and acceptor substituents into the ligand structure directly affects the reduction potential of the Ti (IV) ion in the titanocene structure. Most of the acceptor substituents reduce the reduction potential for ~0.3 V. Since the mechanism of the cytotoxic action of titanocene includes the reduction of Ti^+4^ to Ti^+2^, it is the acceptor substituents (-F, poly-F, -N_3_, etc.) that can be considered as the most promising in the design of further cytotoxic titanocene derivatives.

All compounds studied in the framework of this work showed greater resistance to hydrolysis in aqueous media compared to the starting titanocene dichloride. Both titanocene dicarboxylate containing acceptor substituents (**2e**) and donor substituents (**2b**) have a half-life time in water of approximately 3 h; however, the hydrolysis of more acceptor ligands in the case of **2e** is slower.

Based on the combination of electrochemical, structural, and aqueous stability data, it can be concluded that the most promising compounds for further modification and the study of biological properties are titanocene derivatives with bidentate ligands containing acceptor substituents in the *para*-position to the carboxyl group, for example, those based on 5-substituted salicylic acids.

The series of titanocene dicarboxylates obtained in this work was studied for cytotoxicity on the MCF7 and MCF7-10A cell lines; however, none of the compounds showed an IC50 of less than 100 μM.

## Figures and Tables

**Table 1:** Conditions optimization for the synthesis of titanocene dicarboxylate **2a**.

Entry	Solvent ^1^	T, °C	Time	Yield	Result
1	Acetone	25	2 h	Traces	Destruction
2	Dioxane	25	2 h	15%	Low conversion
3	Methanol	25	2 h	0%	Destruction
4	THF	25	2 h	10%	Low conversion
5	DCM	25	2 h	15%	Partial decomposition
6	Benzene	25	2 h	20%	Low conversion
7	Benzene	40	2 h	45%	Low conversion
8	Benzene	60	2 h	30%	Destruction
9	Benzene	40	3 h	60%	Low conversion
10	Benzene	40	4 h	87%	Pure product
11	Benzene	40	8 h	80%	Partial decomposition
12	Benzene	25	24 h	65%	Partial decomposition

^1^ All tested solvents were absolute and oxygen free.

**Table 2:** Geometric parameters (Å, °) for molecular structures **2a**, **2e**, and **2g**.

Compound	Ti-O1, O3	Ti-Cg1, Cg2	O1-Ti-O3	Cg1-Ti-Cg2	Cp1/Cp2 Dihedral
**2a**	1.908 (2)–1.996 (2)	2.05	91.82 (8)	132	49.5
**2e**	1.952 (2)	1.704	88.8 (1)	134.2	48.2
**2g**	1.927 (2)–1.975 (2)	2.061–2.080	87.68–87.80 (8)	132–132.5	49.3

**Table 3:** Electrochemical potentials of compounds **2a**–**g** (C = 1 × 10^−1^ mol L^−1^) in DMF.

№	R in Ligand	E_red_, V
Cp_2_TiCl_2_	-	−0.62, −1.85
**2a**	-H	−0.85, −2.17
**2b**	4-OBn	−0.88, −2.21
**2c**	4-N_3_	−0.78, −2.01
**2d**	3-F	−0.79, −2.05
**2e**	2,3,4,5,6-F	−0.57, −1.81
**2f**	2-OAc	−0.81, −2.11
**2g**	2-O^−^	−0.90 rev

## Data Availability

The data presented in this study are available on request from the corresponding authors.

## Supplementary Materials

The following supporting information can be downloaded at: https://www.mdpi.com/article/10.3390/ijms24043340/s1.

## References

[1] Sung H., Ferlay J., Siegel R.L., Laversanne M., Soerjomataram I., Jemal A., Bray F. (2021). Global Cancer Statistics 2020: GLOBOCAN Estimates of Incidence and Mortality Worldwide for 36 Cancers in 185 Countries. CA Cancer J. Clin..

[2] Wheate N.J., Walker S., Craig G.E., Oun R. (2010). The Status of Platinum Anticancer Drugs in the Clinic and in Clinical Trials. Dalt. Trans..

[3] McWhinney S.R., Goldberg R.M., McLeod H.L. (2009). Platinum Neurotoxicity Pharmacogenetics. Mol. Cancer Ther..

[4] Miller R.P., Tadagavadi R.K., Ramesh G., Reeves W.B. (2010). Mechanisms of Cisplatin Nephrotoxicity. Toxins.

[5] Chen M.-H., Zheng Y., Cai X.-J., Zhang H., Wang F.-X., Tan C.-P., Chen W.-H., Jia L.-N., Mao Z.-W. (2019). Inhibition of autophagic flux by cyclometalated iridium(III) complexes through anion transportation. Chem. Sci..

[6] Zhou J., Kang Y., Chen L., Wang H., Liu J., Zeng S., Yu L. (2020). The Drug-Resistance Mechanisms of Five Platinum-Based Antitumor Agents. Front. Pharmacol..

[7] Guk D.A., Krasnovskaya O.O., Beloglazkina E.K. (2021). Coordination Compounds of Biogenic Metals as Cytotoxic Agents in Cancer Therapy. Russ. Chem. Rev..

[8] Köpf H., Köpf-Maier P. (1979). Titanocene Dichloride—The First Metallocene with Cancerostatic Activity. Angew. Chem. Int. Ed. English.

[9] Kröger N., Kleeberg U.R., Mross K., Edler L., Saß G., Hossfeld D.K. (2000). Phase II Clinical Trial of Titanocene Dichloride in Patients with Metastatic Breast Cancer. Onkologie.

[10] Köpf-Maier P. (1994). Complexes of Metals Other than Platinum as Antitumour Agents. Eur. J. Clin. Pharmacol..

[11] Harstrick A., Schmoll H.J., Poliwoda H., Sass G., Rustum Y. (1993). Titanocendichloride Activity in Cisplatin and Doxorubicin-Resistant Human Ovarian Carcinoma Cell Lines. Eur. J. Cancer.

[12] Christodoulou C.V., Ferry D.R., Fyfe D.W., Young A., Doran J., Sheehan T.M.T., Eliopoulos A., Hale K., Baumgart J., Sass G. (1998). Phase I Trial of Weekly Scheduling and Pharmacokinetics of Titanocene Dichloride in Patients with Advanced Cancer. J. Clin. Oncol..

[13] Korfel A., Scheulen M., Schmoll H.-J., Gründel O., Harstrick A., Knoche M., Fels L., Skorzec M., Bach F., Baumgart J. (1998). Phase I Clinical and Pharmacokinetic Study of Titanocene Dichloride in Adults with Advanced Solid Tumors. Clin. Cancer Res..

[14] Dean J.A. (1999). Lange’s Handbook of Chemistry.

[15] Ravera M., Cassino C., Monti E., Gariboldi M., Osella D. (2005). Enhancement of the Cytotoxicity of Titanocene Dichloride by Aging in Organic Co-Solvent. J. Inorg. Biochem..

[16] Fernández-Vega L., Silva V.A.R., Domínguez-González T.M., Claudio-Betancourt S., Toro-Maldonado R.E., Maso L.C.C., Ortiz K.S., Pérez-Verdejo J.A., González J.R., Rosado-Fraticelli G.T. (2020). Evaluating Ligand Modifications of the Titanocene and Auranofin Moieties for the Development of More Potent Anticancer Drugs. Inorganics.

[17] Ceballos-Torres J., Caballero-Rodríguez M.J., Prashar S., Paschke R., Steinborn D., Kaluerović G.N., Gómez-Ruiz S. (2012). Synthesis, Characterization and in Vitro Biological Studies of Titanocene(IV) Derivatives Containing Different Carboxylato Ligands. J. Organomet. Chem..

[18] Saturnino C., Sirignano E., Botta A., Sinicropi M.S., Caruso A., Pisano A., Lappano R., Maggiolini M., Longo P. (2014). New Titanocene Derivatives with High Antiproliferative Activity against Breast Cancer Cells. Bioorg. Med. Chem. Lett..

[19] Olszewski U., Hamilton G. (2012). Mechanisms of Cytotoxicity of Anticancer Titanocenes. Anticancer Agents Med. Chem..

[20] Basu Baul T.S., Manne R., Tiekink E.R.T. (2018). Crystalline Bis(η 5 -Cyclopentadienyl)Bis(Benzoato/Carboxylato)Titanium(IV) Precursor-Directed Route to Functional Titanium Dioxide Nanomaterials. J. Coord. Chem..

[21] Wang Z.Q., Lu S.W., Guo H.F., Lu Z.R., Zhou Y.K. (1992). Aqueous Phase Synthesis of Dicarboxylic Derivatives of Titanocene. Synth. React. Inorg. Met. Chem..

[22] Zi -Wei G. (2000). Preparation of Titanocene of Substituted Salicylic Complexes in Aqueous Medium and Substituent Effects and Influences of Acidity. Acta Chim. Sin..

[23] Xiao-mei X., Wei-yong S., Guo-rui M., Zhengzhi Z. (1999). Preparation and Characterization of Dicyclopentadienyl Compound of Titanium(IV)I:—Preparation AndCharacterization of the Dicyclopentadienyl Titanium of Salicylic Acid and Aspirin. J. ZheJiang Univ. Ed..

[24] Juzwa M., Rusin A., Zawidlak-Wegrzyńska B., Krawczyk Z., Obara I., Jedliński Z. (2008). Oligo(3-Hydroxybutanoate) Conjugates with Acetylsalicylic Acid and Their Antitumour Activity. Eur. J. Med. Chem..

[25] Kumar P., Gupta A.K., Priya S., Prasad S. (2011). Mixed Ligand Complexes of Alkali Metal Salts of Thiocol with Salicylic Acid, Acetylsalicylic Acid and 2-Hydroxy-3-Naphthoic Acid. Orient. J. Chem..

[26] Biancalana L., Pampaloni G., Zacchini S., Marchetti F. (2018). Synthesis, Characterization and Behavior in Water/DMSO Solution of Ru(II) Arene Complexes with Bioactive Carboxylates. J. Organomet. Chem..

[27] Lucas C.R., Gabe E.J., Lee F.L. (1988). Cyclopentadienyl–Metal Bond Cleavage and Stereospecific Bromination. The Structure of (η-C 5 H 5 ) 2 Ti(OOCPh) 2 and Its Reactions with Bromine. Can. J. Chem..

[28] Payack J.F., Hughes D.L., Cai D., Cottrell I.F., Verhoeven T.R. (2002). Dimethyltitanocene. Org. Synth..

[29] Wu Y., Chen C., Jia G., Zhu X., Sun H., Zhang G., Zhang W., Gao Z. (2014). Salicylato Titanocene Complexes as Cooperative Organometallic Lewis Acid and Brønsted Acid Catalysts for Three-Component Mannich Reactions. Chem. Eur. J..

[30] Wang J., Chen X., Wang X., Zhang W.Q., Sun H.M., Zhang G.F., Wu Y., Gao Z.W. (2016). Synthesis of Air-Stable Mixed Bis-Carboxylate Titanocene Complexes and Their Catalytic Behaviors in Cross-Aldol and Mannich Reactions. Transit. Met. Chem..

[31] Gao Z., Zhang C., Dong M., Gao L., Zhang G., Liu Z., Wang G., Wu D. (2006). Syntheses and Supramolecular Structures of Two 5-Nitrosalicylate Titanocene Complexes. Appl. Organomet. Chem..

[32] Basu Baul T.S., Manne R., Tiekink E.R.T. (2019). Mono- and di-anionic coordination modes of arylazosalicylates in their bis(η5-cyclopentadienyl)titanium(IV) complexes: Syntheses and crystal structures. Inorg. Chim. Acta.

[33] Döppert K., Klein H.P., Thewalt U. (1986). Bis(Benzoato)-Bis(π-Cyclopentadienyl)-Titan(IV): Darstellung in Wässriger Lösung Und Struktur Einer Neuen Modifikation. J. Organomet. Chem..

[34] Edwards D.A., Mahon M.F., Paget T.J., Summerhill N.W. (2001). Bis(η-Cyclopentadienyl)Titanium(IV)—Salicylate System Revisited and the Characterisation of Two 3,5-Di-t-Butylsalicylate Analogues. The Molecular Structure of |Cp2Ti(Sal)|, (Sal = O2CC6H4O2-). Transit. Met. Chem..

[35] Mugnier Y., Moise C., Laviron E. (1981). Electrochemical Studies of Organometallic Compounds. J. Organomet. Chem..

[36] Banerjee R. (2012). Redox outside the box: Linking extracellular redox remodeling with intracellular redox metabolism. J. Biol. Chem..

[37] Jamieson L.E., Jaworska A., Jiang J., Baranska M., Harrisond D.J., Campbell C.J. (2015). Simultaneous intracellular redox potential and pH measurements in live cells using SERS nanosensors. Analyst.

[38] Guk D.A., Krasnovskaya O.O., Moiseeva A.A., Tafeenko V.A., Ul′yanovskii N.V., Kosyakov D.S., Pergushov V.I., Melnikov M.Y., Zyk N.V., Skvortsov D.A. (2021). New Fe–Cu bimetallic coordination compounds based on ω-ferrocene carboxylic acids and 2-thioimidazol-4-ones: Structural, mechanistic and biological studies. Inorg. Chem. Front..

[39] Xu H.-G., Annamadov S., Mokhir A. (2022). 4-Ferrocenylaniline-based ROS-responsive prodrugs with anticancer activity. J. Organomet. Chem..

[40] Toney J.H., Marks T.J. (1985). Hydrolysis chemistry of the metallocene dichlorides M(η5-C5H5)2Cl2, M = titanium, vanadium, or zirconium. Aqueous kinetics, equilibria, and mechanistic implications for a new class of antitumor agents. J. Am. Chem. Soc..

[41] Takechi E., Inubushi I., Otaka K. (1994). Manufacture of Halogenated Metallocenes. Chem. Abstr..

[42] Shi H., Li J., Wang T., Rudolph M., Hashmi A.S.K. (2022). Catalyst- and Additive-Free Sunlight-Induced Autoxidation of Aldehydes to Carboxylic Acids. Green Chem..

[43] Tsymbal S.A., Moiseeva A.A., Agadzhanian N.A., Efimova S.S., Markova A.A., Guk D.A., Krasnovskaya O.O., Alpatova V.M., Zaitsev A.V., Shibaeva A.V. (2021). Copper-Containing Nanoparticles and Organic Complexes: Metal Reduction Triggers Rapid Cell Death via Oxidative Burst. Int. J. Mol. Sci..

