# Supplementary material for: New Titanocene (IV) Dicarboxylates with Potential Cytotoxicity: Synthesis, Structure, Stability and Electrochemistry
Source: Int J Mol Sci. 2023 Feb 7;24(4):3340. doi: 10.3390/ijms24043340 (PMC9964323; doi:10.3390/ijms24043340)
Supplement: Supplementary file 1 [file ijms-24-03340-s001.zip › ijms-2197246-supplementary.pdf]

# New titanocene (IV) dicarboxylates with potential cytotoxicity: synthesis, structure, stability and electrochemistry

**Dmitry A. Guk[1*], Karina R. Gibadullina[1], Roman O. Burlutskiy[1], Kirill G. Pavlov[1], Anna A. Moiseeva[1], Viktor A. Tafeenko[1], Konstantin A. Lyssenko[1], Erik R. Gandalipov[2], Alexander A. Shtil[3,4], and Elena K. Beloglazkina[1,*]**

[1]Chemistry Department, Lomonosov Moscow State University, Leninskie Gory 1/3, Moscow 119991, Russia.
[2]International Institute of Solution Chemistry of Advanced Materials and Technologies, ITMO University, Saint-Petersburg, Russia
[3]Blokhin National Medical Research Center of Oncology, Moscow, Russia
[4]National Research Nuclear University MEPHI, Moscow, Russia
* Correspondence: beloglazki@mail.ru; dmh200949@gmail.com

## Supplementary information

### 1. Preparation of silver salts

**Silver benzoate (1h).** All procedures were performed in the dark. Benzoic acid (0.537 g, 4.40 mmol) was added to a solution of $NaHCO_3$ (0.370 g, 4.40 mmol) in 20 mL of water in a 100 ml beaker, and the mixture was stirred until gas evolution ceased. The solution was filtered through a paper filter and the filter was washed with 20 ml of water. A solution of $AgNO_3$ (0.679 g, 4.00 mmol) in 5 mL of water was added dropwise to the filtrate. The white precipitate was separated by filtration on a glass filter under reduced pressure, washed with water, ethanol, and dried in a stream of air. The resulting white powder was transferred to a flask and dried several times with benzene on a rotary evaporator to remove traces of water. **1h** was obtained as a white powder. Yield: 0.577 g (63%).$^{1}$HNMR (DMSO-d6, 400 MHz, δppm): 7.93 - 8.00 (m, 2H, -CHα-Ar), 7.34 - 7.47 (m, 3H, -CHβ,γ-Ar ).

**Silver acetylsalicylate (1j).** All procedures were performed in the dark. Acetylsalicylic acid (0.757 g, 4.20 mmol) was added to a solution of $NaHCO_3$ (0.353 g, 4.20 mmol) in 20 mL of water in a 100 ml beaker, and the mixture was stirred until gas evolution ceased. The solution was filtered through a paper filter, the filter was washed with 20 ml of water. A solution of $AgNO_3$ (0.646 g, 3.80 mmol) in 5 mL of water was added dropwise to the filtrate. The white precipitate was separated by filtration on a glass filter under reduced pressure, washed with water, ethanol, and dried in a stream of air. The resulting white powder was transferred to a flask and dried several times with benzene on a rotary evaporator to remove traces of water. **1j** was obtained asa white powder. Yield: 0.594 g (42%).$^{1}$H NMR (DMSO-d6, 400 MHz, δ ppm): 7.88 (dd, J=7.67, 1.70 Hz, 1H-CHα-Ar), 7.37 - 7.43 (m, 1H-CHβ-Ar), 7.23 (td, J=7.50, 1.12 Hz, 1H-CHβ’-Ar), 7.02 (dd, J=7.97, 1.01 Hz, 1H-CHγ-Ar), 2.22 (s, 3H, $-CH_3$).

## 2. IR Spectra of p-aminobenzoic and p-azidobenzoic acids

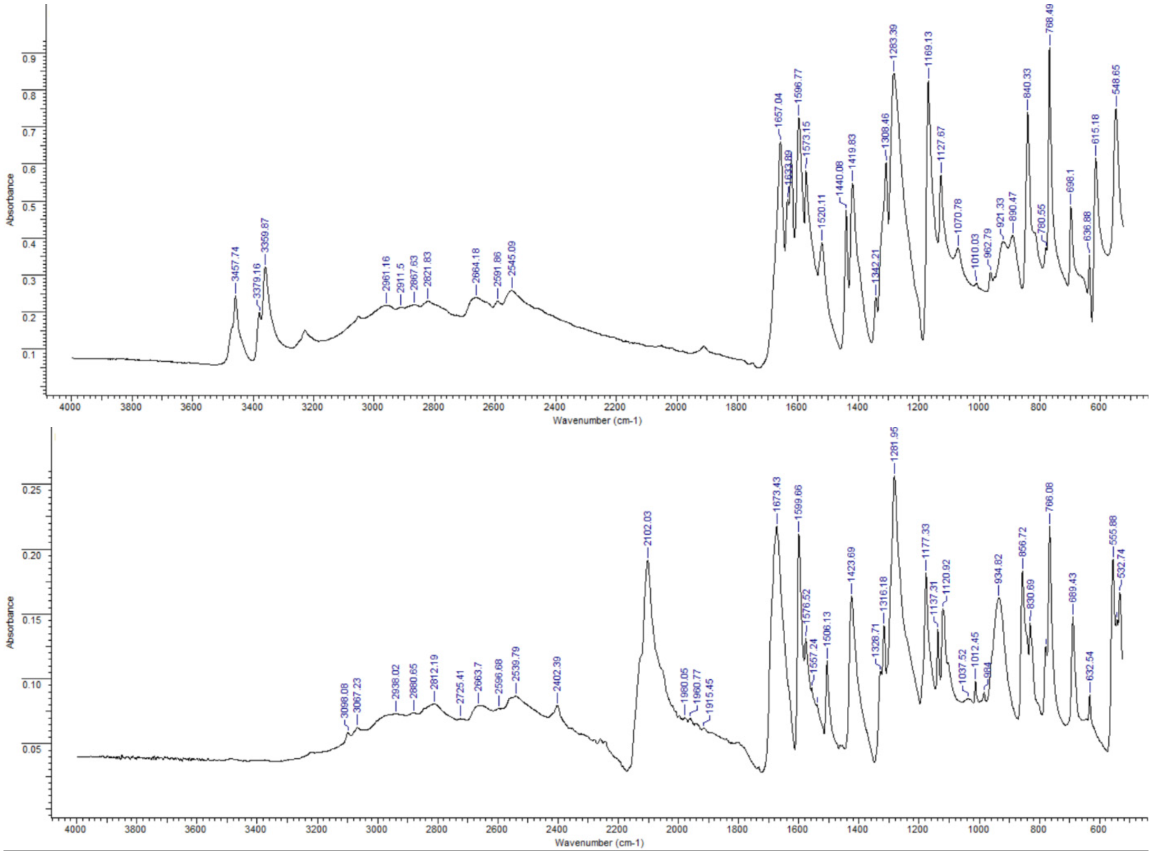


**Figure S1.** IR-Spectra of p-aminobenzoic acid (top) and p-azidobenzoic acid (bottom).

## 2. Selected NMR spectra of titanocene dicarboxylates.

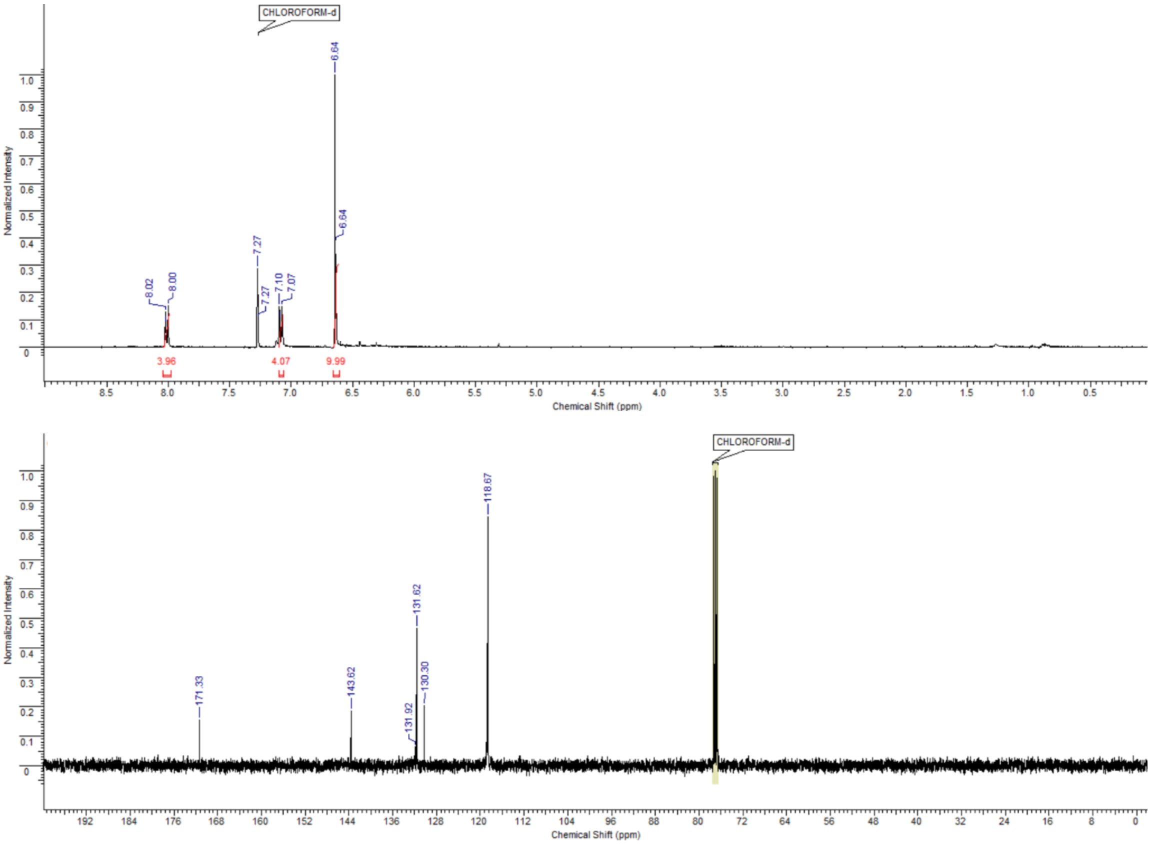


**Figure S2.** $^{1}H$ (top) and $^{13}C$ (bottom) NMR Spectra of compound **2c**.

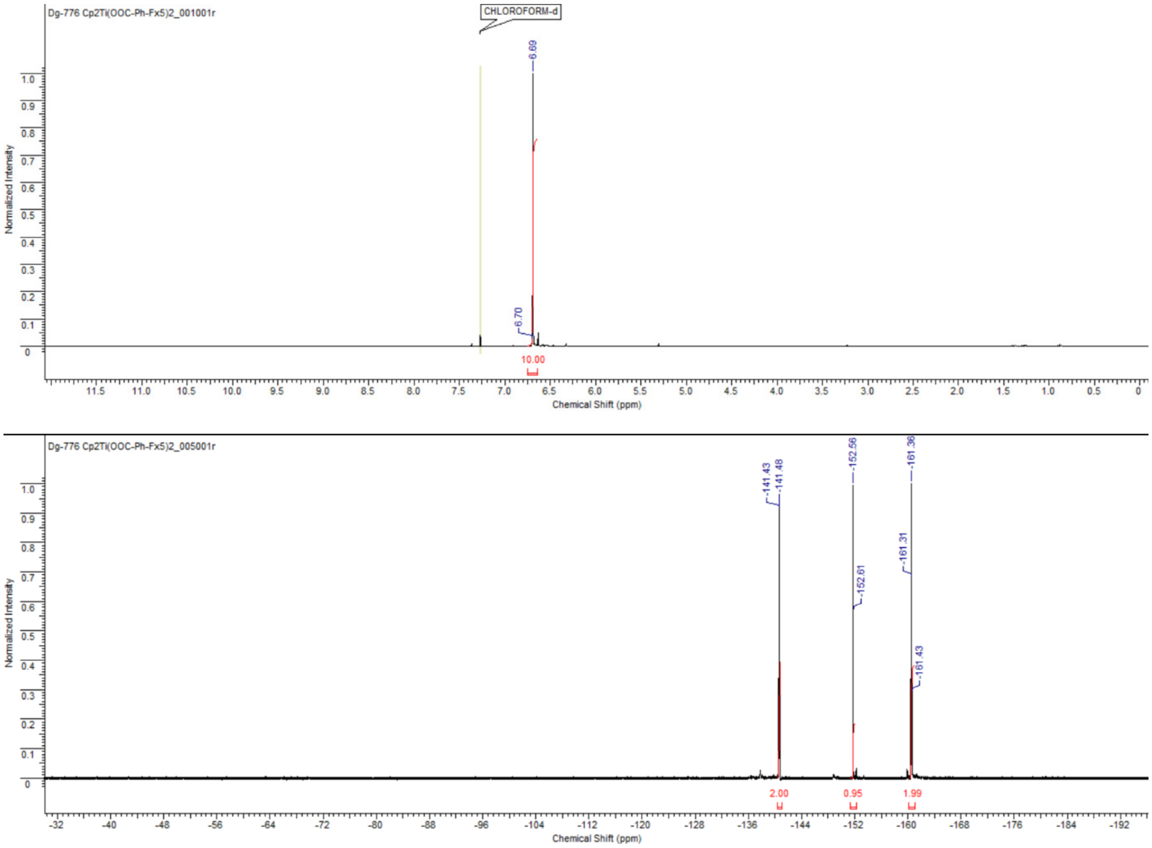


**Figure S3.** $^{1}$H (top) and $^{19}$F (bottom) NMR Spectra of compound **2e**.

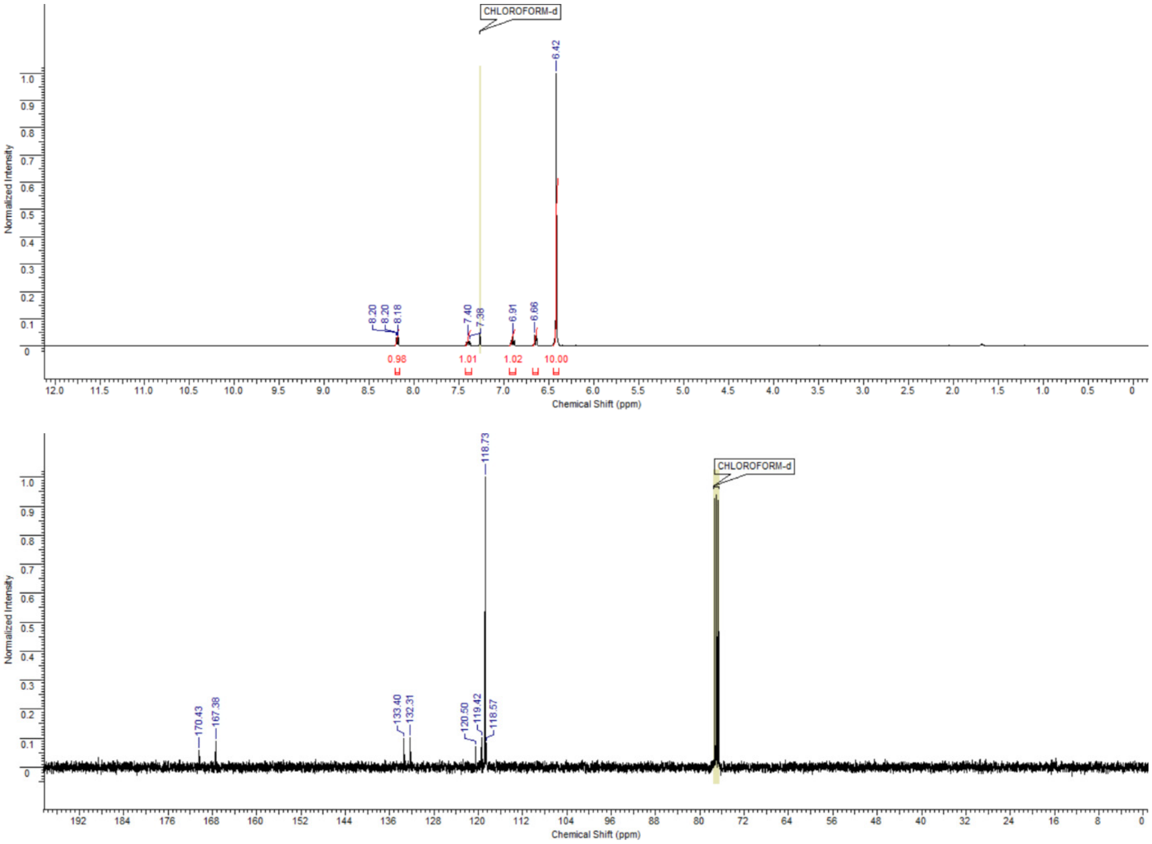


**Figure S4.** $^{1}$H (top) and $^{13}$C (bottom) NMR Spectra of compound **2g**.

## 3. HRMS of compound 2g

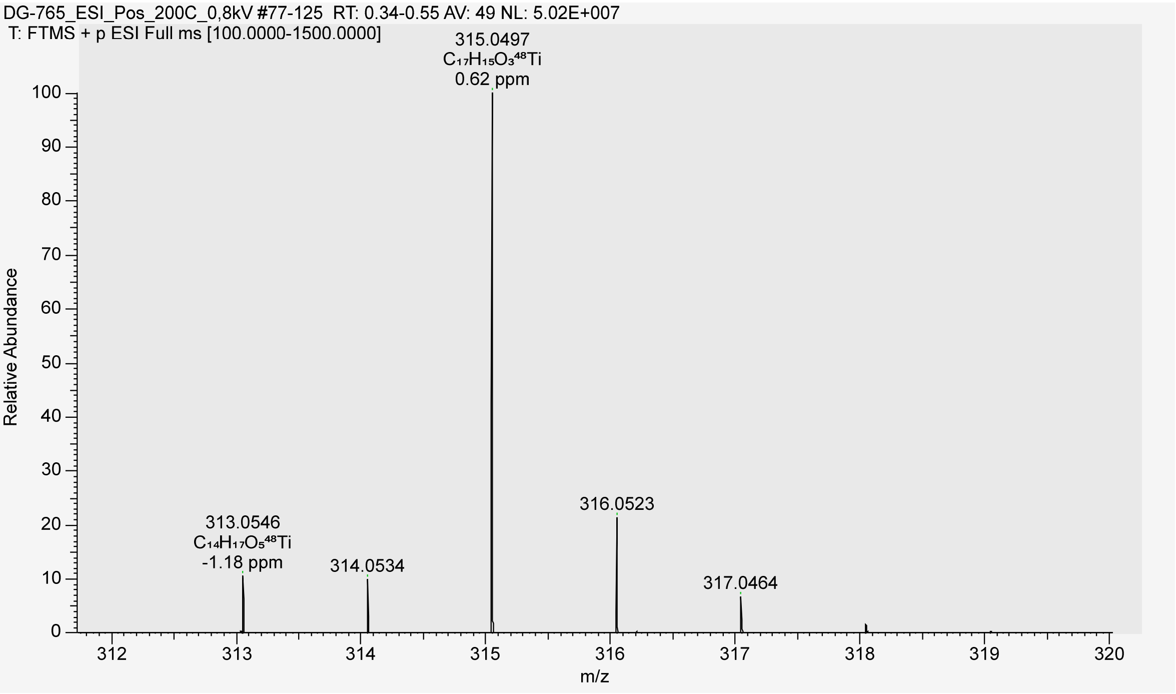


**Figure S5.** HRMS spectrum of compound **2g**.

## 4. CV-curves for titanocene dicarboxylates

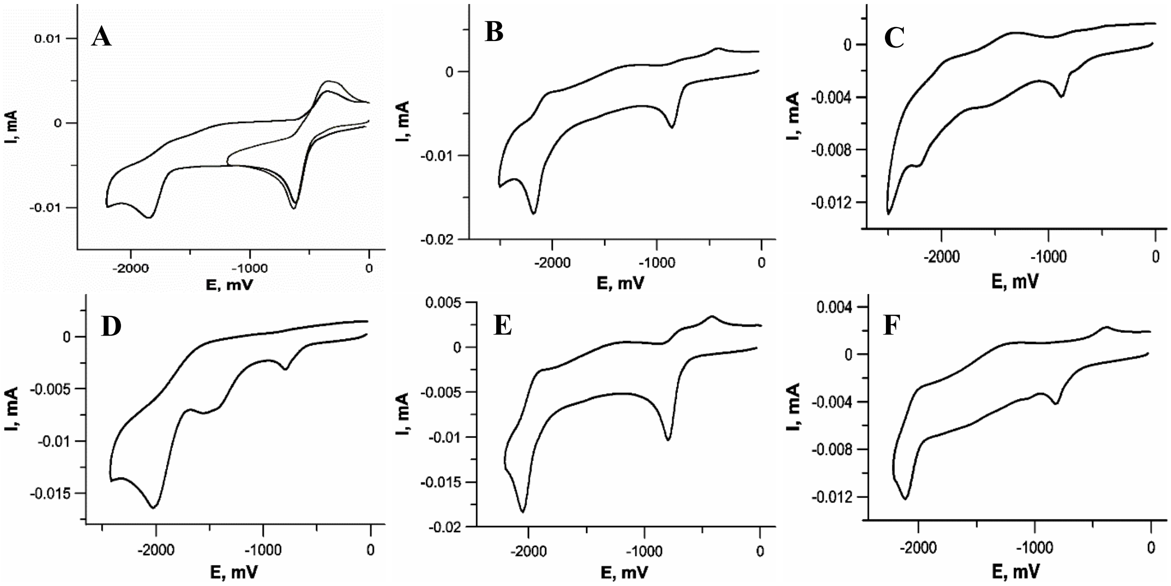


**Figure S6.** CV-curves of titanocene dichloride (A), and compounds **2a** (B), **2b** (C), **2c** (D), **2d** (E), **2f** (F).

## 5. Crystal data and structure refinement for compounds 2a, 2e, 2g

Absorption corrections based on measurements of equivalent reflections were carried out by *SADABS* (multi-scan methods) #. The structures were solved by direct methods with the *SHELXT* program and refined by full matrix least-squares on $F^2$ with *SHELXL*. Positions of all non-H atoms were found from electron difference density maps and refined with individual anisotropic displacement parameters. Positions and individual isotropic displacement parameters for phenyl and cyclopentadienyl H-atoms were positioned geometrically and refined as riding atoms with relative isotropic displacement parameters. Crystal data, data collection and structure refinement details for **2a**, **2e** and **2g** are summarized in Table S1. The structures have been deposited at the Cambridge Crystallographic Data Center with the reference CCDC numbers 2233054,

2233055, 2236508, and they also contain the supplementary crystallographic data. These data can be obtained free of charge from the CCDC *via* http://www.ccdc.cam.ac.uk/data_request/cif

**Table S1.** Crystallographic data for synthesized titanocene derivatives.

| Identification code | **2a** | **2e** | **2g** |
|---|---|---|---|
| Difractomer | Bruker D8 Quest with Photon III detector | Stoe STADI VARI | Bruker D8 Quest with Photon III detector |
| Wavelength, Å | 0.71072 | 1.54186 | 0.71072 |
| Empirical formula | $C_{24}H_{20}O_4Ti$ | $C_{24}H_{10}F_{10}O_4Ti$ | $C_{17}H_{14}O_3Ti$ |
| Formula weight | 420.291 | 600.22 | 314.18 |
| Temperature, K | 110 | 295(2) | 100(2) |
| Crystal system | Orthorhombic | Orthorhombic | Monoclinic |
| Space group | $P2_12_12_1$ | Pnna | $P2_1/c$ |
| Unit cell dimensions | | | |
| *a* (Å) | 7.500(4) | 21.8684(5). | 12.150(2) |
| *b* (Å) | 12.510(5) | 13.0894(4) | 23.537(5) |
| *c* (Å) | 21.126(10) | c 8.0409(2) | 15.390(3) |
| β (°) | 90 | 90 | 108.02(3) |
| Volume | 1982.0(16) $Å^3$ | 2301.66(10) $Å^3$ | 4185.3(16) $Å^3$ |
| Z(Z') | 4(1) | 4 (0.5) | 12 (3) |
| Density (calculated) | 1.408 | 1.732 | 1.496 |
| Absorption coefficient | 4.59 | 4.216 | 6.20 |
| F(000) | 872 | 1192 | 1944 |
| θ range for data collection | 1.89 to 29. | 5.271 to 66.963. | 1.76 to 29°. |
| Reflections collected | 21893 | 15075 | 41278 |
| Independent reflections | 5261 [R(int) = 0.0932] | 2046 [R(int) = 0.0672] | 11105 [$R_{int}$=0.0965] |
| Observed reflections | 4391 | 1325 | 8029 |
| Completeness to $\theta_{max}$ , % | 99.9 | 99.3 | 99.9 |
| Data/parameters | 5261/262 | 2046 / 198 | 11105/568 |
| Goodness-of-fit on $F^2$ | 1.051 | 0.977 | 1.051 |
| Final R indices [I>2sigma(I)] | R1=0.0509, wR2=0.1034 | R1 = 0.0421, wR2 = 0.1148 | R1=0.0589, wR2=0.1462 |
| R indices (all data) | R1 = 0.0656, wR2 = 0.1118 | R1 = 0.0661, wR2 = 0.1279 | R1= 0.0840, wR2= 0.1277 |
| Largest diff. peak and hole | 0.4557 and -0.6502 e.Å $^{-3}$ | 0.217 and -0.343 e.Å $^{-3}$ | 0.740 and -0.622 e.Å $^{-3}$ |

## 6. Stability in aqueous media studies

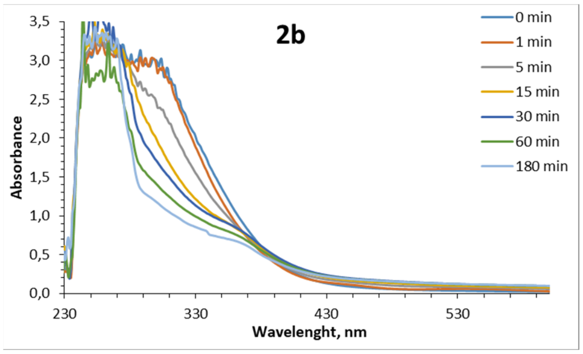


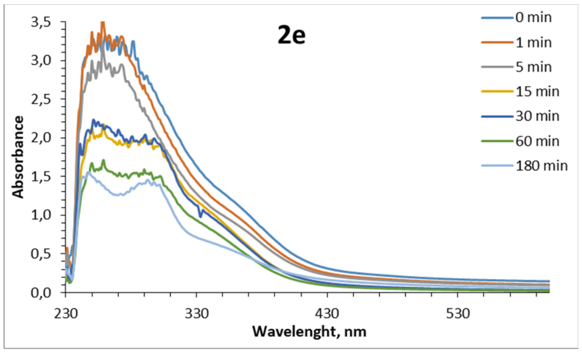

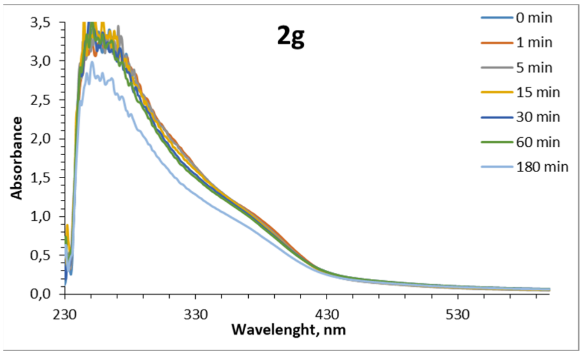


**Figure S7.** Full absorbtion spectra of compounds **2b** (top left), **2e** (top right), **2g** (bottom), initial concentration = 0.4 mM obtained in water with addition of 10% per volume of DMSO, after different time intervals.
